# Community meal services for older adults and healthy aging: a structural equation modeling study in Nanjing, China

**DOI:** 10.3389/fpubh.2026.1934658

**Published:** 2026-09-21

**Authors:** Siying Wang, Yiming Yao, Shuxin Dong, Huanhuan Liu, Haibo Zhang, Xunke Wang, Junlong Shen, Jie Li

**Affiliations:** School of Health Economics and Management, Nanjing University of Chinese Medicine, Nanjing, China

**Keywords:** chain service utilization, community senior canteens, healthy aging, silver economy, structural equation modeling

## Abstract

**Objective:**

Community senior canteens have become an important component of community-based meal services and healthy aging strategies in China. However, insufficient alignment between service provision and the diverse needs of older adults may constrain service utilization and long-term sustainability. This study aimed to examine the associations among factors related to older adults’ use of community senior canteens and to identify patterns of behavioral association that may inform the optimization of community meal services.

**Methods:**

A community-based cross-sectional survey was conducted among adults aged 60 years and older across the 11 administrative districts of Nanjing, China. Using a combination of stratified sampling, quota sampling, and snowball sampling, 1,568 complete cases were included in the final SEM analysis. An adapted SICAS framework was used to construct a structural equation model (SEM) to examine the sequential relationships among awareness, interaction, purchase, and sharing. Reliability, validity, and model fit were evaluated before structural path analysis.

**Results:**

The measurement and structural models demonstrated satisfactory fit. All three hypothesized associations were statistically significant (*p* < 0.001). Awareness was positively associated with Interaction, Interaction was positively associated with Purchase, and Purchase was positively associated with Sharing.

**Conclusion:**

Older adults’ use of community senior canteens was characterized by a pattern of positive associations among awareness, interaction, purchase, and sharing. Given the cross-sectional design, these findings should not be interpreted as evidence of temporal or causal relationships. The observed associations may nevertheless provide useful evidence for improving service communication, user engagement, and service quality in community meal services.

## 1 Introduction

Population aging has become one of the most profound demographic transformations of the twenty-first century, fundamentally reshaping public health systems, social security, and long-term care worldwide (1). According to the United Nations World Population Prospects 2024 medium-variant projection, the global population aged 65 years and older is projected to exceed 1.6 billion by 2050, accounting for approximately 16% of the world’s total population (1). This unprecedented demographic transition presents substantial challenges to sustainable economic and social development. In response, the World Health Organization (WHO) introduced the concept of Healthy Aging, emphasizing that healthy aging extends beyond disease prevention and medical care to encompass the maintenance of functional ability, independence, and social participation throughout later life (2, 3).

In recent years, community meal services have become an integral component of community long-term care systems in many countries, including the United States, Japan, the United Kingdom, and several Nordic countries (4). Growing evidence suggests that these services not only improve nutritional status and food security among older adults but also reduce social isolation, strengthen social connectedness, and facilitate aging in place, thereby contributing to healthy aging. As population aging continues to accelerate, an increasing number of countries have recognized the silver economy as an important strategy for addressing aging-related challenges while promoting sustainable economic development. Accordingly, community meal services have gradually evolved from traditional welfare programs into integrated service models that simultaneously generate social value and economic sustainability, providing new opportunities for building resilient community-based care systems for older adults. Against this global backdrop, China is experiencing one of the largest and most rapid demographic transitions in the world. The continuous growth of the older population has substantially increased the demand for care, healthcare, and long-term care services for older adults, while simultaneously positioning the silver economy as an emerging driver of economic growth (5–8).

Although community meal services have become an important topic in aging research worldwide, considerable differences exist in their development across countries. In Europe and North America, research has primarily focused on the public health benefits of community meal services, including improvements in nutritional health, social participation, and reductions in healthcare utilization (9, 10). In contrast, Japan has placed greater emphasis on integrating community meal services into local care networks for older adults and community governance systems (11). These studies provide valuable evidence for improving community-based care for older adults and demonstrate that community meal services have become an effective public health intervention for promoting healthy aging (12).

Compared with many developed countries, China has continuously strengthened its response to population aging by promoting the integration of community-based and home-based care services for older adults, with community meal assistance becoming an increasingly important component of the care system for older adults (13). Meanwhile, declining household size, increasing numbers of empty-nest households, and weakening family caregiving capacity have intensified the dietary care needs of older adults, particularly those who are living alone, living with disabilities, or of advanced age (14, 15). Under these circumstances, community senior canteens have evolved into an important platform linking families, communities, and care services for older adults. Beyond providing nutritional support and food safety, they also help alleviate family caregiving burdens, encourage social participation, and support active and healthy aging.

As one of China’s economically developed metropolitan cities and one of the earliest cities to enter a deeply aging society, Nanjing represents a typical setting for investigating community meal service. In recent years, the city has continuously expanded its community meal service network by establishing a diversified service delivery system involving government agencies, market actors, and social organizations. Innovative practices, including central kitchens combined with community meal delivery and smart meal assistance, have been widely implemented, substantially improving service accessibility, operational efficiency, and resource integration. These developments provide a valuable practical context for examining the operational mechanisms of community senior canteens (16–18).

Despite the continuous expansion of service provision, community senior canteens still face several challenges, including insufficient identification of heterogeneous needs among older adults, mismatches between service provision and actual demand, limited operational sustainability, and inadequate market vitality. Therefore, understanding the determinants of older adults’ willingness to use community senior canteens and optimizing service provision under the framework of the silver economy have become critical issues for promoting the high-quality development of community-based care services for older adults (19–23).

Nanjing was selected as the study setting because of its representativeness in terms of population aging, the development of community-based care services for older adults, and the advancement of the silver economy. Therefore, Nanjing provides an appropriate setting for the present study (24–26). This study focuses on community senior canteens in Nanjing and employs a structural equation model (SEM) based on questionnaire data collected from older adults to systematically examine the key determinants of older adults’ willingness to use community senior canteens and to test the relationships among these determinants. The findings are expected to contribute to the literature on older adults’ use of community meal services in the context of the silver economy by providing empirical evidence for optimizing service provision, improving resource allocation and operational efficiency, and informing policies aimed at promoting the high-quality development of community-based care services for older adults.

## 2 Participants and methods

### 2.1 Participants

This study employed a community-based cross-sectional survey conducted in Nanjing, Jiangsu Province, China, between October and November 2025. The study population comprised adults aged 60 years and older residing in the 11 administrative districts of Nanjing. The survey assessed older adults’ awareness of community meal services, utilization patterns, dining behaviors, dining scenarios, and open-ended feedback. As the direct users of community meal services, older adults are best positioned to provide firsthand information regarding key aspects of service quality, including food taste, nutritional and health value, the convenience of dine-in and meal delivery services, and age-friendly environmental design. Participants were eligible for inclusion if they met the following criteria: (1) were aged ≥60 years; (2) were able to understand the questionnaire and voluntarily agreed to participate in the study; and (3) were able to complete the questionnaire independently or, if necessary, with assistance from a caregiver according to their own responses and intentions.

### 2.2 Methods

#### 2.2.1 Survey instrument

A structured questionnaire was used for data collection. The questionnaire was developed based on the findings of semi-structured interviews conducted with 21 older adults, 20 adult children or primary caregivers, and 9 community senior canteen operators or community workers from the target population. In addition, focus group discussions were conducted to identify the core needs of participants across different age groups, family structures, and service-use scenarios. The interview and focus group findings adequately reflected the diverse perspectives of service users, family decision-makers, and service providers regarding community senior canteens in Nanjing. Based on these findings, together with relevant policy documents, community meal service policies, and the SICAS theoretical framework, the questionnaire was developed (27).

The questionnaire consisted of five sections: (1) sociodemographic characteristics, including gender, age, educational level, pension income, living arrangement, and mobility status; (2) awareness and utilization of community senior canteens, including awareness channels, previous use of community senior canteens, and overall dining experiences, to assess participants’ awareness of and attitudes toward community senior canteens; (3) utilization characteristics of community senior canteens, including dining mode, dining time, frequency of use, meal price, payment method, and the main reasons for choosing community senior canteens; (4) the SICAS scale, comprising four dimensions: Awareness, Interaction, Purchase, and Share; and (5) open-ended questions, which collected participants’ suggestions and comments regarding community senior canteens.

To ensure the appropriateness of the questionnaire design, a pilot survey was conducted prior to the formal investigation. A total of 78 pilot questionnaires were randomly distributed online. Based on the issues identified during the pilot survey, the questionnaire was revised item by item, and the validity of the returned questionnaires was subsequently evaluated.

#### 2.2.2 Data collection

A multi-stage sampling strategy was adopted to improve geographic coverage while maintaining the feasibility of field-based data collection. The 11 administrative districts of Nanjing were first treated as geographic strata, and quotas were allocated according to the population size of each district. Within the district-level quotas, participants were recruited through snowball sampling from eligible older adults.

A pilot survey was first conducted online, and the questionnaire was revised according to problems identified during the pretest, including ambiguous wording and inappropriate response options. The formal survey was subsequently conducted using paper questionnaires. The formal survey was conducted using paper-based questionnaires through face-to-face recruitment in community senior canteens. For older adults who experienced difficulties completing the questionnaire because of age-related visual or operational limitations, adult children or caregivers could provide assistance with the participant’s informed consent. Importantly, assistants were instructed to record the participant’s own responses rather than provide their own evaluations or preferences.

A total of 1,698 questionnaires were distributed, of which 1,678 were returned, yielding a response rate of 98.82%. After systematic quality screening based on predefined data-cleaning criteria, 28 invalid questionnaires were excluded, leaving 1,650 questionnaires for further analysis. Among these, 1,568 complete cases were included in the final SEM analysis after excluding cases with missing values on the model variables. Thus, the final analytical sample comprised 1,568 older adults.

#### 2.2.3 Reliability and validity

The results of the formal survey demonstrated satisfactory internal consistency. Cronbach’s α coefficients for the four SICAS dimensions (Awareness, Interaction, Purchase and Sharing) were 0.872, 0.808, 0.873, and 0.721, respectively. All Cronbach’s α coefficients exceeded the recommended threshold of 0.70, indicating good internal consistency of the measurement scale.

The Kaiser–Meyer–Olkin (KMO) test was performed to evaluate the suitability of the data for factor analysis. The KMO value was 0.942, indicating good structural validity and excellent sampling adequacy for factor analysis. Subsequently, confirmatory factor analysis (CFA) was conducted to further assess the construct validity of the measurement model.

#### 2.2.4 Statistical analysis

Structural equation modeling (SEM) was selected as the primary analytical approach because the proposed framework involves multiple theoretically related latent constructs—Awareness, Interaction, Purchase, and Sharing—and examines their interrelationships simultaneously. Unlike traditional multiple linear regression, which generally treats predictors as directly observed variables and typically evaluates one dependent relationship at a time, SEM allows latent constructs to be represented by multiple observed indicators while explicitly accounting for measurement error (27). SEM also enables the assessment of the measurement model through confirmatory factor analysis (CFA) and the structural model within a unified analytical framework. This approach was therefore considered appropriate for simultaneously evaluating the reliability and validity of the adapted SICAS constructs and testing the hypothesized associations among them. The methodological advantages of SEM over conventional regression analysis have also been discussed in previous comparative research.

Data were analyzed using SPSS and AMOS 28.0. Structural equation modeling (SEM) was employed to examine the associations among the four latent constructs and to evaluate the hypothesized structural model. Model fit was assessed using the chi-square to degrees of freedom ratio (χ^2^/df), the Comparative Fit Index (CFI), the Incremental Fit Index (IFI), the Root Mean Square Error of Approximation (RMSEA), the Relative Fit Index (RFI), and the Tucker–Lewis Index (TLI). The significance of path coefficients was assessed using *p* values, with *p* < 0.05 considered statistically significant. Standardized coefficients were reported to facilitate interpretation of the relative strength of associations (28, 29).

### 2.3 Ethics

This study employed an anonymous questionnaire survey and did not collect any personally identifiable information. Before completing the questionnaire, all participants were informed of the purpose of the study, participated voluntarily, and were free to withdraw at any time. This study was a non-interventional survey and did not involve any procedures that posed potential risks or harm to the participants. According to the regulations of the authors’ affiliated institution, formal ethical approval was not required for this type of anonymous, non-interventional research.

## 3 Model construction

### 3.1 Theoretical model

The SICAS framework was selected as the theoretical basis because its central proposition is particularly relevant to service-utilization contexts in which users move from initial perception and information processing to interaction, behavioral action, and post-use sharing. Originally developed to characterize consumer behavior in an increasingly interactive and connected environment, the SICAS framework comprises five stages: Sense, Interest and Interactive, Connect and Communicate, Action, and Share. Unlike traditional linear consumer behavior models, SICAS emphasizes the role of user engagement, bidirectional communication, and post-use sharing in behavioral conversion.

To investigate the behavioral pathways underlying older adults’ use of community senior canteens, this study developed a structural equation model based on an adapted SICAS framework. Community senior canteen utilization is a service decision that involves not only an individual’s recognition of service availability but also the acquisition and evaluation of information, communication with service providers or family members, actual service use, and subsequent evaluation or recommendation. These characteristics correspond conceptually to the major behavioral stages represented by SICAS.

Based on this contextual adaptation, the original five-stage framework was operationalized into four latent constructs. First, “Sense” was conceptualized as Awareness, referring to older adults’ recognition and understanding of community senior canteens and their perceived value. Second, “Interest and Interactive” and “Connect and Communicate” were combined into Interaction, because both stages represent users’ active engagement with service-related information, communication, and participation, and the distinction between them was not sufficiently meaningful in the context of community senior canteens. Third, “Action” was operationalized as Purchase, representing actual or intended utilization-related behavior, such as dining at the canteen, convenient payment, and continued use. Finally, “Share” was retained as Sharing, reflecting users’ willingness to evaluate, recommend, or communicate their service experiences to others.

Accordingly, a four-stage behavioral association model (Awareness–Interaction–Purchase–Sharing) was established (Figure 1), and the 13 items of the Q23 scale were classified into four latent constructs to examine whether community senior canteen users’ behavioral transitions exhibit significant sequential transmission effects.

**Figure 1:**
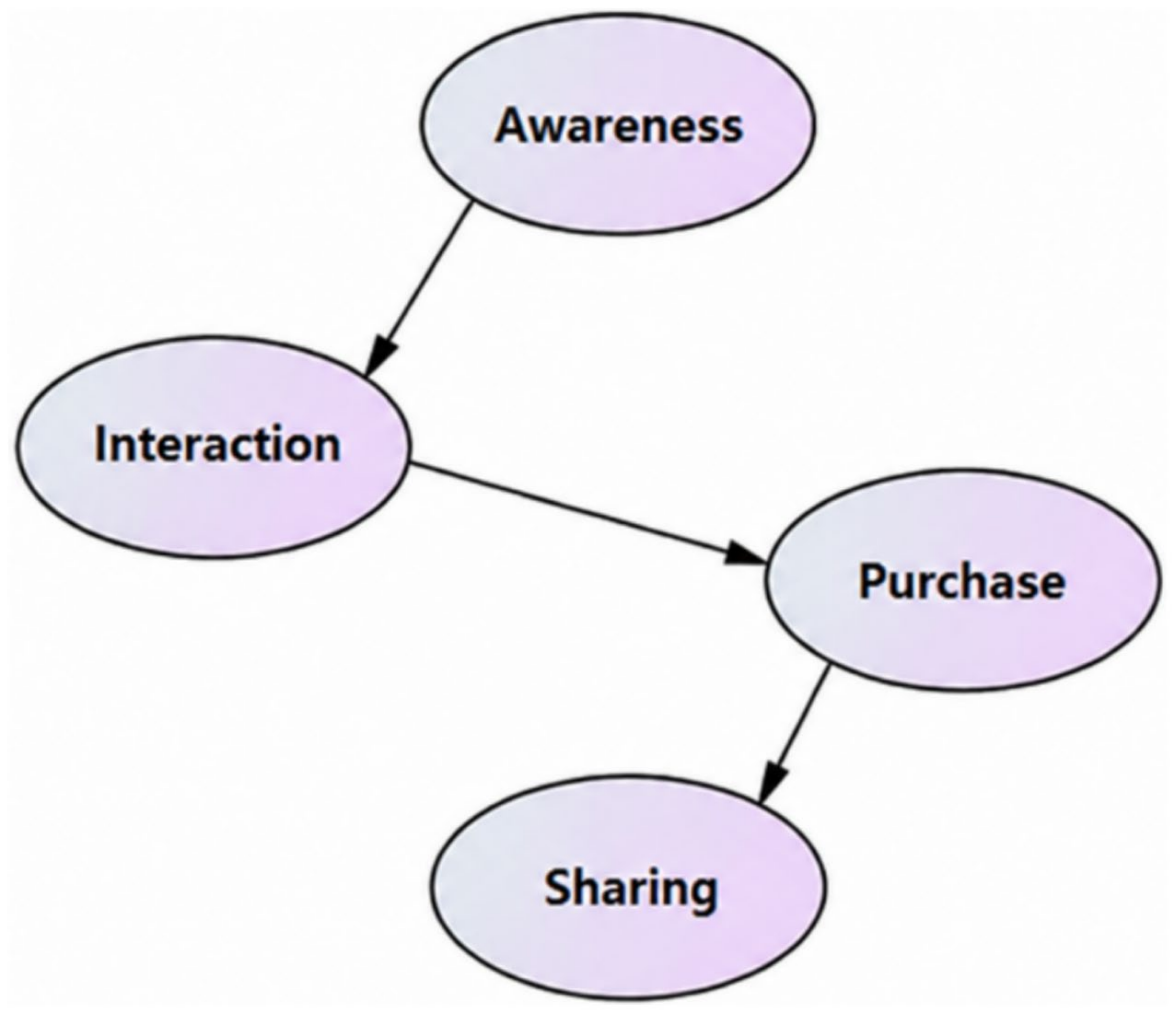
Four-stage chain model of awareness, interaction, purchase, and sharing.

### 3.2 Model estimation

#### 3.2.1 Measurement model assessment

The measurement model demonstrated a satisfactory overall fit to the data, with all fit indices meeting the recommended criteria. As shown in Table 1, with all fit indices meeting the recommended criteria. Specifically, the χ^2^/df (CMIN/DF) was 1.404, the root mean square error of approximation (RMSEA) was 0.029, the relative fit index (RFI) was 0.944, the incremental fit index (IFI) was 0.988, and the Tucker–Lewis index (TLI) was 0.983, indicating that the proposed measurement model was appropriate for subsequent analyses.

**Table 1:** Results of confirmatory factor analysis.

Indicator	Reference criteria	Observed results
CMIN/DF	1–3 Excellent, 3–5 Good	1.404
RMSEA	< 0.05 Excellent, < 0.10 Good	0.029
RFI	> 0.90 Excellent, > 0.80 Good	0.944
IFI	> 0.90 Excellent, > 0.80 Good	0.988
TLI	> 0.90 Excellent, > 0.80 Good	0.983

After establishing an adequate measurement model fit, the convergent validity and composite reliability of the latent constructs were further evaluated. The CFA model of the behavioral intention scale is presented in Figure 2. As shown in Table 2, all constructs exceeded the recommended thresholds for average variance extracted (AVE) and composite reliability (CR), demonstrating satisfactory convergent validity and internal consistency. As shown in Table 3, all item-to-construct paths were statistically significant (*p* < 0.001), with standardized loadings exceeding the 0.5 threshold. This confirms that the items are valid measures for the four dimensions: Awareness, Interaction, Purchase, and Sharing.

**Figure 2:**
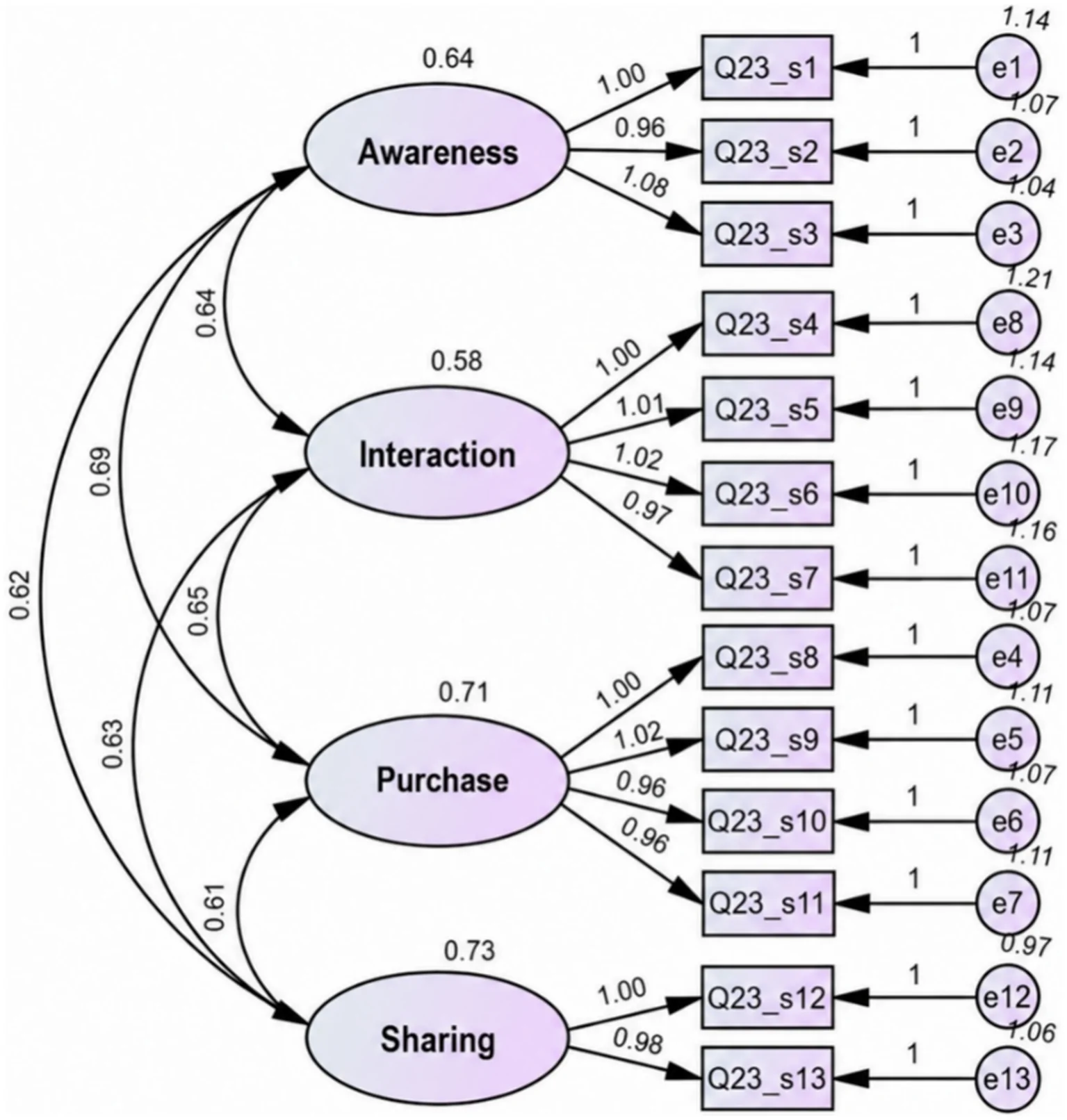
Confirmatory factor analysis (CFA) model diagram.

**Table 2:** Measurement model: convergent validity and composite reliability.

Item	Path relationship	Construct	Estimate	AVE	CR
Q23_s1	←	Awareness	0.799	0.661	0.854
Q23_s2	←	Awareness	0.794		
Q23_s3	←	Awareness	0.845		
Q23_s4	←	Interaction	0.751	0.531	0.819
Q23_s5	←	Interaction	0.734		
Q23_s6	←	Interaction	0.718		
Q23_s7	←	Interaction	0.710		
Q23_s8	←	Purchase	0.871	0.727	0.914
Q23_s9	←	Purchase	0.788		
Q23_s10	←	Purchase	0.883		
Q23_s11	←	Purchase	0.866		
Q23_s12	←	Sharing	0.804	0.668	0.801
Q23_s13	←	Sharing	0.830		

**Table 3:** Measurement model: standardized factor loadings.

Item	Path relationship	Construct	Estimate
Q23_s1	←	Awareness	0547
Q23_s2	←	Awareness	0.554
Q23_s3	←	Awareness	0.583
Q23_s4	←	Interaction	0.539
Q23_s5	←	Interaction	0.568
Q23_s6	←	Interaction	0.584
Q23_s7	←	Interaction	0.546
Q23_s8	←	Purchase	0.632
Q23_s9	←	Purchase	0.588
Q23_s10	←	Purchase	0.584
Q23_s11	←	Purchase	0.587
Q23_s12	←	Sharing	0.634
Q23_s13	←	Sharing	0.613

#### 3.2.2 Structural model evaluation

Following validation of the measurement model, the structural model was further evaluated. As shown in Figure 3 and Table 4, the structural model demonstrated a satisfactory overall fit. Specifically, the χ^2^/df was 1.551, RMSEA was 0.034, RFI was 0.938, IFI was 0.982, and TLI was 0.977. All model fit indices satisfied the recommended criteria, indicating that the proposed four-stage sequential structural model showed a satisfactory fit to the observed data.

**Figure 3:**
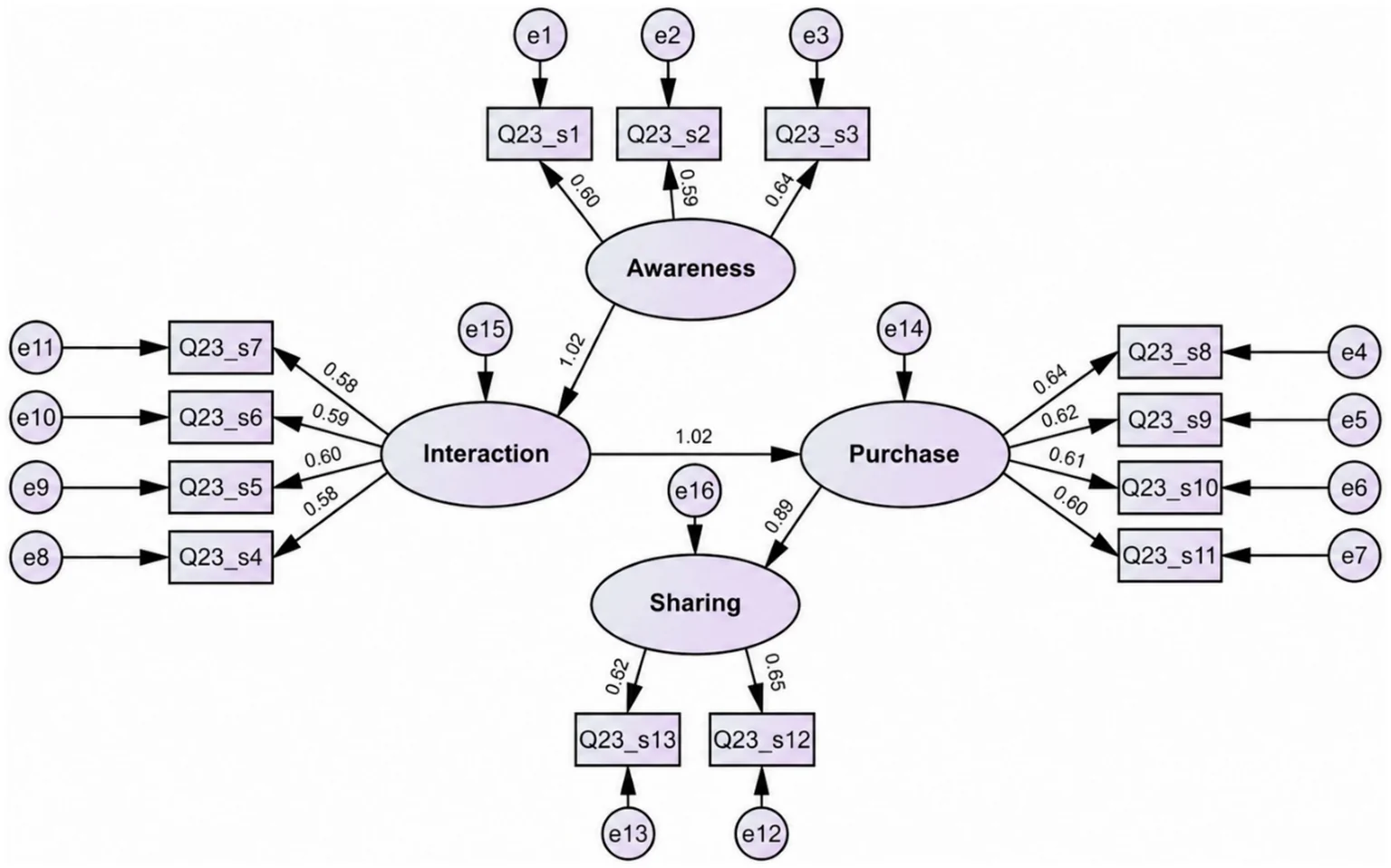
Four-stage behavioral association model.

**Table 4:** Goodness-of-fit indices for the four-stage chain structural model.

Indicator	Reference criteria	Observed results
CMIN/DF	1–3 Excellent, 3–5 Good	1.551
RMSEA	< 0.05 Excellent, < 0.10 Good	0.034
RFI	> 0.90 Excellent, > 0.80 Good	0.938
IFI	> 0.90 Excellent, > 0.80 Good	0.982
TLI	> 0.90 Excellent, > 0.80 Good	0.977
CFI	≧ 0.95 Excellent,≧ 0.90 Good	0.996
GFI	≧0.905Excellent,≧ 0.90 Good	0.989
AGFI	≧ 0.90 Excellent,≧ 0.80 Good	0.982

## 4 Results

### 4.1 Demographic characteristics of the study participants

As shown in Table 5, a total of 1,568 older adults participated in the survey. Of these, 51.3% were male and 48.7% were female. Participants aged 65–74 years accounted for more than half of the sample (53.1%). Most had a junior or senior high school education (58.5%) and a monthly income of RMB 3,001–8,000 (56.1%). Nearly half lived with their spouse (45.3%), 50.8% could travel independently, and 51.0% were proficient in smartphone use. Additionally, 38.5% reported being eligible for or receiving meal subsidies.

**Table 5:** Demographic characteristics of participants (*N* = 1,568).

Characteristic	Number of participants	Percentage (%)
Gender		
Male	805	51.3
Female	763	48.7
Age		
60–64 years	389	24.8
65–69 years	418	26.7
70–74 years	414	26.4
≥75 years	347	22.1
Educational level		
Primary school or below	308	19.6
Junior high schoo	463	29.5
Senior high school/technical secondary school	454	29.0
Junior college	276	17.6
Bachelor’s degree or above	67	4.3
Monthly income		
≤ RMB 3,000	337	21.5
RMB 3,001–5,000	469	29.9
RMB 5,001–8,000	411	26.2
RMB 8,001–10,000	264	16.8
≥ RMB 10,001	87	5.5
Living situation		
Living alone	417	26.6
living with spouse	710	45.3
Living with children	441	28.1
Ease of movement		
Convenient (can travel independently)	796	50.8
Moderate (some difficulty traveling)	462	29.5
Inconvenient (mostly requires assistance from others)	310	19.8
Whether eligible for or receiving meal subsidies or related benefits		
Yes	603	38.5
No	721	46.0
not sure	244	15.6
Level of proficiency in using smartphones		
Proficient (can use QR code payments, view WeChat/short video content)	800	51.2
Average (can use QR code payments or basically view information)	503	32.1
Not very skilled (needs assistance from others)	159	10.1
Cannot do it	106	6.8

### 4.2 Descriptive statistics and distributional characteristics

As shown in Table 6, the mean scores of the 13 measurement items ranged from 3.07 to 3.23, slightly above the midpoint of the 5-point Likert scale (3.00). Awareness and purchase had relatively higher scores, while interaction had the lowest. Skewness ranged from −0.28 to −0.09 and kurtosis from 1.77 to 1.91, with all values within the recommended thresholds for approximate normality (absolute skewness < 3; absolute kurtosis < 8).

**Table 6:** Descriptive statistics and distributional characteristics of the measurement items.

Construct	Measurement item	Item description	Mean	SD	Skewness	Kurtosis
Awareness	Q23_s1	Consumption trust	3.16	1.33	−0.15	1.77
	Q23_s2	Price transparency	3.12	1.29	−0.19	1.90
	Q23_s3	Nutritional matching	3.20	1.33	−0.22	1.81
Interaction	Q23_s4	Information awareness	3.07	1.34	−0.11	1.80
	Q23_s5	Activity participation	3.10	1.30	−0.15	1.83
	Q23_s6	Contact channels	3.11	1.33	−0.14	1.81
	Q23_s7	Communication accessibility	3.14	1.30	−0.14	1.85
Purchase	Q23_s8	Consumption convenience	3.23	1.30	−0.28	1.91
	Q23_s9	Payment convenience	3.16	1.37	−0.24	1.78
	Q23_s10	Continued use	3.16	1.32	−0.20	1.84
	Q23_s11	Future frequency of use	3.10	1.33	−0.09	1.81
Sharing	Q23_s12	Satisfaction sharing	3.15	1.30	−0.20	1.87
	Q23_s13	Recommendation to relatives/friends	3.12	1.33	−0.23	1.86

### 4.3 Multicollinearity assessment

As shown in Table 7, the tolerance values ranged from 0.422 to 1.000, while the VIF values ranged from 1.000 to 2.372. All values were within the commonly accepted thresholds, indicating that no substantial multicollinearity was present among the predictor variables.

**Table 7:** Multicollinearity diagnostics of the predictor variables.

Dependent variable	Predictor	Tolerance	VIF
Interaction	Awareness	1.000	1.000
Purchase	Awareness	0.534	1.874
	Interaction	0.534	1.874
Sharing	Awareness	0.439	2.276
	Interaction	0.437	2.288
	Purchase	0.422	2.372

### 4.4 Structural path analysis

Structural path analysis was conducted to examine the hypothesized associations among the four latent constructs. The structural model showed satisfactory overall fit, with χ^2^/df = 1.551, RMSEA = 0.034, RFI = 0.938, IFI = 0.982, and TLI = 0.977.

All three hypothesized adjacent associations were statistically significant. Awareness was positively associated with Interaction, Interaction was positively associated with Purchase, and Purchase was positively associated with Sharing. The measurement-model results, including factor loadings and their significance tests, are presented in Table 8, whereas the structural path results are presented in Table 9. Overall, the results were consistent with the hypothesized associations among Awareness, Interaction, Purchase, and Sharing.

**Table 8:** Measurement model: Item loadings and significance.

Item	Path relationship	Construct	Estimate	S. E.	CR	*p*
Q23_s1	←	Awareness	0.600	0.201	10.989	***
Q23_s2	←	Awareness	0.594	0.177	10.981	***
Q23_s3	←	Awareness	0.644	0.092	11.689	***
Q23_s4	←	Interaction	0.575	0.145	11.882	***
Q23_s5	←	Interaction	0.596	0.122	10.880	***
Q23_s6	←	Interaction	0.594	0.135	11.686	***
Q23_s7	←	Interaction	0.578	0.122	10.372	***
Q23_s8	←	Purchase	0.641	0.142	11.540	***
Q23_s9	←	Purchase	0.622	0.185	12.085	***
Q23_s10	←	Purchase	0.609	0.212	11.754	***
Q23_s11	←	Purchase	0.600	0.082	11.687	***
Q23_s12	←	Sharing	0.661	0.164	10.298	***
Q23_s13	←	Sharing	0.624	0.218	11.106	***

“***” indicates *p* < 0.001.

**Table 9:** Results of structural path analysis for the four-stage chain structural equation model.

Path relationship	Unstandardized estimate	S. E.	CR	*p*
Awareness → Interaction	1.024	0.068	15.149	***
Interaction → Purchase	0.970	0.062	15.754	***
Purchase → Sharing	1.031	0.063	16.320	***

“***” indicates *p* < 0.001.

## 5 Discussion

### 5.1 Key findings

This study identified a consistent pattern of positive associations among Awareness, Interaction, Purchase, and Sharing among older adults using community senior canteens. Awareness was positively associated with Interaction, Interaction with Purchase, and Purchase with Sharing. These findings were consistent with the hypothesized ordering of the four constructs, although the cross-sectional design does not permit conclusions regarding temporal or causal relationships.

One possible explanation is that greater awareness may be associated with lower perceived uncertainty, while greater interaction may be associated with higher confidence and perceived accessibility. Similarly, favorable utilization-related experiences may be associated with greater satisfaction and willingness to share information.

These findings may have several practical implications for community senior canteen management. Service strategies could be organized around the four behavioral domains identified in the present study. At the awareness stage, providers could communicate core service attributes such as food safety, price transparency, and nutritional suitability through accessible channels (30). At the interaction stage, accessible menu information, nutritional guidance, community activities, and convenient communication channels may strengthen engagement with potential users (31, 32). At the utilization stage, attention to meal quality, service attitude, payment convenience, and accessibility may improve the user experience. Finally, mechanisms that facilitate feedback and voluntary recommendation may help strengthen the sharing dimension. These recommendations should be regarded as practice-oriented implications derived from the observed associations rather than as experimentally demonstrated causal interventions (7, 33–35).

### 5.2 Comparison with international research

In recent years, the focus of international research on meal assistance services for older adults has shifted from basic support to technology-enabled solutions and sustainable development; this trend offers a meaningful contrast to the practical explorations of community senior canteens in China.

At the level of technology application, French research—based on the AGGIR framework for assessing older adults’ dependency—systematically examined the acceptance of smart meal assistance systems. Its core findings indicate that whether technology can effectively enhance service efficiency depends heavily on the accurate identification of older adults’ functional decline and cognitive characteristics, rather than the simple transplantation of technological solutions (36). Research from Spain and other countries further points out that while Internet of Things (IoT) devices can replace some manual care functions in areas such as route planning and nutritional balancing, risks such as privacy infringement, data security, and the digital divide are becoming structural barriers to the implementation of these technologies. This warning reveals the inherent tension between the logic of efficiency and the vulnerability of the older population (37).

In terms of service organization models, Japan’s meal delivery services have evolved through four stages—emergence, inception, adjustment, and optimization. Their core experience lies in matching diverse dining scenarios—such as home meal delivery, centralized community meal service, and family-style dining—to the varying levels of mobility among older adults, thereby achieving a refined alignment between service provision and individual functional status. In contrast, while China’s community senior canteens have achieved rapid expansion of their facility networks under policy impetus, current research is largely limited to policy narratives and descriptive accounts of operational challenges. There remain significant shortcomings in the precision of identifying heterogeneous user needs, the mechanistic analysis of behavioral decision-making pathways, and the quantitative empirical testing of segmentation strategies (38–41).

Against this background, the present study complements existing research by focusing on the behavioral associations underlying community senior canteen utilization, rather than primarily examining service delivery models or technological implementation. This perspective may help explain how older adults move from awareness and interaction to utilization-related behavior and sharing.

### 5.3 Impact on health policy and practice

Meal assistance services for older adults should be integrated into broader strategies for the integration of medical care, care for older adults, and nutrition, as well as community health promotion. Given the importance of perceived service attributes, including food safety, price transparency, and nutritional suitability, regulatory authorities should strengthen the existing institutional compliance framework by adopting a risk-based inspection approach (42–45). More targeted and rigorous supervision should be implemented for meal service providers serving vulnerable populations, such as the oldest-old and older adults with disabilities, to improve food safety protection (46, 47).

Beyond ensuring food safety, meal assistance services should also strengthen their health promotion function. As the observed associations span awareness, interaction, utilization-related behavior, and sharing, health promotion strategies should be embedded throughout the service process (32). This includes improving the transparency of nutritional information, integrating nutritional screening and health assessments into meal service settings, and promoting the community-wide diffusion of healthy eating behaviors through continuous improvements in service experience (48–50).

In addition, a collaborative governance mechanism involving governments, communities, healthcare institutions, and social organizations should be established to support coordinated provision of both basic and personalized services. Such efforts would facilitate the transformation of meal assistance services from a model focused primarily on food safety and nutrition toward a comprehensive health promotion approach characterized by precise service matching and person-centered care (51, 52).

Taken together, these findings suggest that community meal services should evolve from a food assistance model toward an integrated community health service that combines nutritional support, health promotion, and social participation (53–55).

### 5.4 Limitations

This study has several limitations. Although stratified and quota sampling ensured coverage across Nanjing’s 11 municipal districts, the use of snowball sampling may have introduced selection bias and limited generalizability. The cross-sectional design and self-reported data limit causal inference and may be subject to self-report and common method bias. Caregiver-assisted questionnaire completion may have also introduced response bias, although caregivers were instructed to report participants’ own responses. In addition, discriminant validity was not independently assessed using established criteria such as the Fornell–Larcker criterion or HTMT, and potentially relevant factors, including socioeconomic status and previous service experience, were not included in the model. Future studies should adopt longitudinal and multi-site designs with probability-based sampling to further validate the proposed framework.

## 6 Conclusion

Based on 1,568 valid questionnaires from older adults across 11 districts in Nanjing, this study developed and empirically examined a four-stage behavioral association model—Awareness → Interaction → Purchase → Sharing—using structural equation modeling. The results showed significant positive associations between each pair of adjacent constructs. The findings were consistent with the hypothesized association pattern, although causal and temporal relationships cannot be established because of the cross-sectional design.

## Data Availability

The raw data supporting the conclusions of this article will be made available by the authors, without undue reservation.
