# Supplementary material for: Community meal services for older adults and healthy aging: a structural equation modeling study in Nanjing, China
Source: Front Public Health. 2026 Sep 21;14:1934658. doi: 10.3389/fpubh.2026.1934658 (PMC13637669; doi:10.3389/fpubh.2026.1934658)
Supplement: Supplementary file 1 [file Data_Sheet_1.pdf]

03013 Questionnaire (English Version)

Survey on Service Demand and Evaluation of Community Senior Canteens in Nanjing

Thank you for taking the time to complete our questionnaire. We are graduate students from XXX University, currently conducting market research on community senior canteens in Nanjing. This questionnaire aims to understand the diverse needs and usage preferences regarding community senior canteens in Nanjing, in order to propose more targeted service and promotional recommendations. Please answer based on your actual situation. All responses will be kept anonymous and the data collected will only be used for internal analysis. Please feel free to fill in the questionnaire honestly. Your genuine responses will provide valuable data for our research. Thank you very much for your support and cooperation!

Note: Community senior canteens refer to senior canteens, meal assistance points, or community canteens that provide meal services for residents within the community. The term "seniors" in this questionnaire mainly refers to residents aged 60 and above. If some seniors have difficulty filling out the questionnaire, their children may assist them.

I. Basic Information

1. Your gender [Single choice]

- A. Male
- B. Female

2. Your age [Single choice]

- A. 60–64 years old
- B. 65–69 years old
- C. 70–74 years old
- D. 75 years old and above

3. Your education level [Single choice]

- A. Primary school or below
- B. Junior high school

- C. Senior high school / Technical secondary school
- D. College diploma
- E. Bachelor's degree or above

4. Your approximate monthly retirement pension [Single choice]

- A. ¥3,000 and below
- B. ¥3,001–5,000
- C. ¥5,001–8,000
- D. ¥8,001–10,000
- E. ¥10,001 and above

5. Your living situation [Single choice]

- A. Living alone
- B. Living with spouse
- C. Living with children
- D. Other (please specify: ____)

6. Your mobility level [Single choice]

- A. Good (can travel independently)
- B. Fair (some difficulty traveling)
- C. Poor (mostly need assistance from others)

7. Do you receive meal subsidies or have related qualifications? [Single choice]

- A. Yes
- B. No
- C. Not sure

8. How well can you use a smartphone? [Single choice]

- A. Proficient (can scan QR codes for payment, use WeChat/watch short videos)
- B. Fair (can scan QR codes for payment or read basic information)
- C. Not very well (need assistance from others)
- D. Cannot use at all

II. Awareness and Usage of Community Senior Canteens

9. Have you heard of community senior canteens? [Single choice]

- A. Yes

- B. No (skip to Question 16)

10. How did you mainly learn about community senior canteens? [Single choice]

- A. Community/neighborhood committee notification
- B. Introduction from friends and family
- C. WeChat groups/Official accounts
- D. Saw while passing by
- E. Short videos/media reports
- F. Other (please specify: ____)

11. Have you ever used a community senior canteen? [Single choice]

- A. Yes
- B. No (skip to Question 16)

12. How do you mainly contact the community senior canteen? [Multiple choice]

- A. Community announcements/phone notifications
- B. On-site consultation (inquire at the canteen/ask while passing by)
- C. Consult via WeChat groups/official accounts
- D. Children/family members relay or handle on my behalf
- E. I have not contacted the community senior canteen yet

13. Approximately how long does it take to walk from your home to the community senior canteen? [Single choice]

- A. Within 5 minutes
- B. 6–10 minutes
- C. 11–15 minutes
- D. 16 minutes and above

14. When you eat at the community senior canteen, who decides and who pays? [Single choice]

- A. I decide, I pay
- B. I decide, children/family members pay
- C. Children/family members decide, I pay
- D. Children/family members decide, children/family members pay
- E. Other (please specify: ____)

15. Based on your actual dining experience, please rate your satisfaction with the following aspects of the community senior canteen [Matrix scale question]

(1 = Very Dissatisfied, 2 = Dissatisfied, 3 = Neutral, 4 = Satisfied, 5 = Very Satisfied)

	Items	1	2	3	4	5
Satisfaction Rating	Food Hygiene and Safety					
	Food Taste					
	Nutritional Health (low oil, low salt, sugar control, etc.)					
	Transparent and Affordable Pricing (value for money)					
	Senior-friendly Environment (seating, circulation, restrooms, etc.)					
	Queueing and Meal Pickup Efficiency					
	Service Attitude					
	Distance and Travel Convenience					

III. Usage Behavior and Scenarios of Community Senior Canteens

If you have not heard of or used community senior canteens before, please consider the following questions based on what you would do if you were to use a community senior canteen in the future.

Note: Community senior canteens refer to senior canteens, meal assistance points, or community canteens that provide meal services for residents within the community.

16. If you were to choose a community senior canteen, please rate the importance of the following aspects [Matrix scale question]

(1 = Very Unimportant, 2 = Unimportant, 3 = Neutral, 4 = Important, 5 = Very Important)

	Items	1	2	3	4	5
Importance Rating	Food Hygiene and Safety					
	Food Taste					
	Nutritional Health (low oil, low salt, sugar control, etc.)					
	Transparent and Affordable Pricing (value for money)					
	Senior-friendly Environment (seating, circulation, restrooms, etc.)					
	Queueing and Meal Pickup Efficiency					

	Service Attitude					
	Distance and Travel Convenience					

17. After learning about community senior canteens, which dining method would you prefer? [Single choice]

- A. Dine-in
- B. Takeout
- C. Delivery/pickup by others
- D. Not fixed

18. Which meal times would you most likely use at the community senior canteen? [Multiple choice]

- A. Breakfast
- B. Lunch
- C. Dinner
- D. Not fixed

19. Your current or expected frequency of using community senior canteens [Single choice]

- A. No more than 2 times per month
- B. 1–2 times per week
- C. 3–5 times per week
- D. Almost every day

20. Acceptable price per meal at the community senior canteen [Single choice]

- A. ¥10 and below
- B. ¥10–15
- C. ¥15–20
- D. Above ¥20

21. Which payment method would you most likely use at the community senior canteen? [Single choice]

- A. Cash
- B. Card payment

- C. Mobile payment
- D. Family members pay on my behalf

22. What is the most important reason for choosing a community senior canteen? [Single choice]

- A. Affordable price
- B. Healthy and nutritious
- C. Convenient and time-saving
- D. Hygienic and safe
- E. Suitable taste
- F. Social companionship

23. Based on your own situation, please rate the following statements about community senior canteens [Matrix scale question]

(1 = Strongly Disagree, 2 = Disagree, 3 = Neutral, 4 = Agree, 5 = Strongly Agree)

Dimension	Statements	1	2	3	4	5
Brand and User Perception	I trust the food safety and quality of community senior canteens					
	I think community senior canteens have transparent pricing and good overall value					
	I believe the nutritional composition of community senior canteens is					

	more suitable for seniors					
Interest and Interaction	I would like to learn about new dishes/discounts/events at community senior canteens					
	I would like to participate if community senior canteens offer health lectures, holiday activities, etc.					
Connection and Communication	I prefer to contact or get information from community senior canteens through familiar channels (e.g., community notifications, WeChat groups, phone calls, etc.)					

	I find the feedback channels of community senior canteens smooth (can easily provide opinions and get responses)					
Action and Conversion	I find it convenient to eat there (distance, scheduling, pickup, etc.)					
	I find ordering/payment (including family payment) not troublesome					
	In the next month, I am willing to continue using or try community senior canteens					
	I am willing to increase my usage frequency (e.g., from occasional to weekly)					

Experience and Sharing	I am satisfied with the overall service of community senior canteens					
	I am willing to recommend community senior canteens to friends and family					

24. If community senior canteens could improve/add services, which would you most like to see prioritized? [Multiple choice]

- A. Nutrition labeling/healthy meals (low oil, low salt, sugar control, etc.)
- B. Faster pickup/advance ordering available
- C. Home delivery/pickup service
- D. Family/friends packages (to share with family)
- E. Lower prices or easier subsidy access
- F. Social activities (holiday events, interest groups, etc.)
- G. Other (please specify: ____)

25. Do you have any other suggestions or ideas for community senior canteens? ________________ [Fill in the blank]

（注：部分内容可能由 AI 生成）
